# Semantic Segmentation of the Choroid in Swept Source Optical Coherence Tomography Images for Volumetrics

**DOI:** 10.1038/s41598-020-57788-z

**Published:** 2020-01-23

**Authors:** Shingo Tsuji, Tetsuju Sekiryu, Yukinori Sugano, Akira Ojima, Akihito Kasai, Masahiro Okamoto, Satoshi Eifuku

**Affiliations:** 1 The Department of Cellular and Integrative Physiology, Fukushima Medical University, Fukushima, Japan; 2 The Department of Ophthalmology, Fukushima Medical University, Fukushima, Japan; 3 The Department of Systems Neuroscience, Fukushima Medical University, Fukushima, Japan

**Keywords:** Diagnostic markers, Tomography

## Abstract

The choroid is a complex vascular tissue that is covered with the retinal pigment epithelium. Ultra high speed swept source optical coherence tomography (SS-OCT) provides us with high-resolution cube scan images of the choroid. Robust segmentation techniques are required to reconstruct choroidal volume using SS-OCT images. For automated segmentation, the delineation of the choroidal-scleral (C-S) boundary is key to accurate segmentation. Low contrast of the boundary, scleral canals formed by the vessel and the nerve, and the posterior stromal layer, may cause segmentation errors. Semantic segmentation is one of the applications of deep learning used to classify the parts of images related to the meanings of the subjects. We applied semantic segmentation to choroidal segmentation and measured the volume of the choroid. The measurement results were validated through comparison with those of other segmentation methods. As a result, semantic segmentation was able to segment the C-S boundary and choroidal volume adequately.

## Introduction

The choroid is the tissue with high blood flow per unit volume in the body, and the choriocapillaris that is located in the innermost layer of the choroid nourishes both the photoreceptor and the retinal pigment epithelium^1,2^. The emergence of OCT technology has been one of the most significant advances in ophthalmology in the past 20 years^3–5^. In the era of OCT, the choroidal thickness has become a useful parameter in the diagnosis of diseases associated with the choroid, such as Vogt-Koyanagi-Harada disease^6^, choroidal tumors^7^, age-related macular degeneration (AMD)^8,9^, and central serous chorioretinopathy (CSC)^10^. In the pachychoroid spectrum, which includes CSC and part of AMD, it has been suggested that localized choroidal thickening may be involved in local serous retinal detachment or choroidal neovascularization^11,12^. In order to detect such regional structural changes of the choroid, a choroidal thickness map and three-dimensional (3D) analysis may be useful^13^. Manual choroidal segmentation in each slice is indeed very useful^14,15^, but that of the volume scan for the choroidal thickness map is time-consuming work, and may not be practical in a clinic. The problem with automatic segmentation of the choroid is that the C-S boundaries are difficult to detect because they are ill-defined on the OCT images. Several methods, Graph-cut segmentation^16,17^ or segmentation based on the gradient of pixels^18^, have been proposed previously. At present, automated segmentation may not have the same accuracy as manual segmentation.

Recently, an artificial neural network represented a significant breakthrough, and deep learning architectures have been applied to various fields, such as computer vision, bioinformatics, and medical image analysis^19^. Semantic segmentation, based on the convolutional neural network, is a novel image analysis technique^20,21^, describes the process of associating each pixel of an image with a class label, and is used in the fields of autonomous driving and medical imaging analysis. This method is potentially useful for choroidal segmentation in 3D choroidal analysis. To our knowledge, there has only been one report describing the segmentation of the choroid using a convolutional neural network, in which the spectral domain OCT image was used^22^. The wide range of depth penetration in SS-OCT has the advantage of visualizing the choroid. In the current study, we investigated semantic segmentation of the choroid in SS-OCT images of the choroidal thickness map or the volume analysis. We also examined the similarities and reproducibility of the automated semantic segmentation using a deep convolutional neural network (DCNN), and other methods of choroidal segmentation.

## Subjects and Methods

### Informed consent

This prospective, noncomparative case-series was approved by the Institutional Ethics Committee of Fukushima Medical University, and was conducted in accordance with the tenets of the Declaration of Helsinki of 1975, as revised in 2000. Informed consent was obtained from all individuals participating in this study.

Subjects: 43 eyes from 34 healthy individuals were analyzed. 8 males and 26 females, mean age: 28 (range: 22–44) y.o., mean axial length: 24.84 (range: 22.79–26.20) mm. SS-OCT images from 6 eyes of 3 individuals were used as baseline data. 290 randomly selected images in the cube scans of 37 eyes from 31 individuals were used to analyze similarities. The twenty-five eyes were scanned two times in a row. The two volume images in each eye were used to examine reproducibility between these two images.

### Image acquisition

SS-OCT (Plex Elite 9000-TM, Carl Zeiss Meditec, Inc., Dublin, California, USA) 6 × 6-mm (a 500 × 500 sampling in one volume scan) OCT images of the healthy eyes were acquired using OCT angiography mode between November 1, 2018 and March 24, 2019, after application of mydriatics (5 mg/ml tropicamide and 5 mg/ml phenylephrine sodium chloride, Santen Pharmaceutical Co. Ltd., Osaka, Japan). Two ophthalmic imaging experts took SS-OCT images. The SS-OCT instrument has a central wavelength of 1,060 nm, a bandwidth of 100 nm, A-scan depth of 3.0 mm in tissue, full-width (at half-maximal axial resolution) of about 5 µm in tissue, and lateral resolution at the retinal surface of about 20 µm. The FastTrac motion-correction software built into the SS-OCT was used during image acquisition. We used the structural OCT data only for the analysis.

### Semantic segmentation

**Figure 1:**
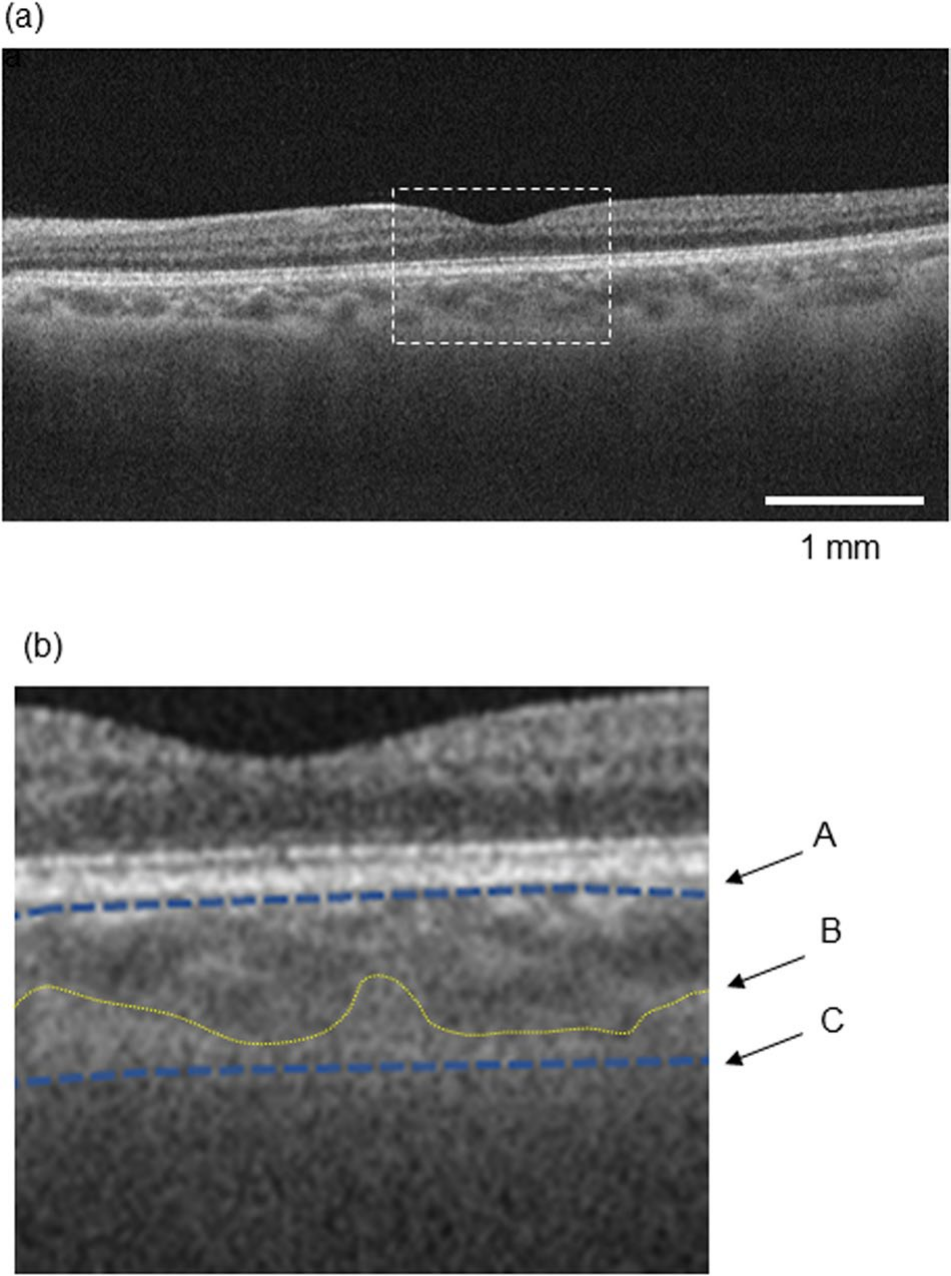
Definition of labeling. An original swept source OCT image (**a**). The area in the dashed line square is magnified in (**b**). The upper limit of the choroid was just beneath the retinal pigment epithelium (A) when the image was labeled for training. The choroid scleral (C-S) boundary was defined along the extended low contrast line between the choroid and the sclera (C), not the outer boundary of the large choroidal vessel (B).

Three thousand B-scan images from six cube scans of six eyes were used as the ground truth data for training. The labeling of the choroidal boundary on the SS-OCT images was carried out by one retina specialist (Y.S.) manually (Fig. 1). The C-S boundary was defined along the extended low contrast line between the choroid and the sclera, not the outer boundary of the large choroidal vessel. The area of the choroid was defined as the area between the line beneath the retinal pigment epithelium and the C-S boundary on a B-scan image. We approached choroidal segmentation using the SegNet, which was developed by members of the Computer Vision and Robotics Group at the University of Cambridge, UK, based on the VGG19 network, primarily designed for large-scale natural image classification and transfer learning. This network consisted of 16 convolutional layers, with pooling as the encoder and three upsampling transposed convolutional layers as the decoder^23^. This decoder was trained to convert the features from the encoder to class labels, accompanying skip layers being used to process higher resolution features from the lower layers.

In the VGG19 network, the final layer uses a softmax function to produce a probability output. The area ratio of the choroidal area to the outside of the choroidal area on the images was about 1:9 in an SS-OCT image in the present study, so we balanced the classes using class weighting. For adapting this network to various SS-OCT images, we augmented the training images with translation and rotation. Stochastic gradient descent with momentum was used for training this network, with a momentum of 0.9, a learning rate of 0.001, and mini-batch size to use for a prediction of 1. We found that this network converged within 100 epochs.

### C-S boundary segmentation

A cubic smoothing spline was applied to determine the C-S boundary. The cubic smoothing spline *f* minimizes

$$p\mathop{\sum }\limits_{j=1}^{n}{|{y}_{j}-f({x}_{j})|}^{2}+(1-p)\int {|{D}^{2}f(t)|}^{2}dt$$

where *n* represents the number of entries of *x*, and the integral is over the smallest interval containing the whole entries of *x*. In addition, *x*_*j*_ and *y*_*j*_ refer to the *j*th entries of *x* and *y*, respectively, and *D*^2^*f* represents the second order derivative of the function *f*. In the present study, the smoothing parameter *p* was 0.01. The number was selected for smoothing the edges of the C-S boundary correctly and keeping its details.

Graph cut segmentation was performed as described previously by Chiu, S. *et al*.^24^.

### Validation of similarities

The Sørensen–Dice coefficient (DSC) among the segmentations performed with the SegNet and other methods (the graph cut and manual segmentation) was calculated to evaluate similarities in 290 SS-OCT B scan images randomly selected from 37 eyes of 31 individuals. Three retina specialists (A.O., A.K., T.S.) segmented the choroid independently using the same method as that used for ground truth labeling; they enclosed the area of the choroid in a polygonal shape using a personal computer. The following equation was used to calculate the DSC.

$$DSC=\frac{2|A\cap B|}{|A|+|B|}$$

*A* and *B* are the choroidal region segmented by either method respectively.

### Validation of feasibility and reproducibility

To evaluate the feasibility and reproducibility of the SegNet model, we measured the intraclass correlation coefficient (ICC) between the repeatedly measured choroidal volumes of twenty-five eyes. The volumetric data were calculated from the SS-OCT cube images. We registerd the center of the fovea in the two images taken in row using ImageJ (version 1.52n)^25^ along the x-, y-, and z-axes. We cropped the original 3D choroidal volume at sizes of 250 × 500 × 500 pixels (Height × Width x Depth) to 170 × 450 × 400 pixels to remove the outside of the overlapping area.

### Visualization of choroidal thickness

The choroidal thickness was plotted as a heat map to visualize the regional choroidal thickening^8,26^, and was converted using the following normalization method:

$$Y=(M-m)\frac{X-{x}_{min}}{{x}_{max}-{x}_{min}}$$

*X*: the original choroidal thickness measured after segmentation.

*Y*: the choroidal thickness for 64 color heat map.

*x*_*max*_: maximum value of the original choroidal thickness in the total scan area.

*x*_*min*_: minimum value of the original choroidal thickness in the total scan area.

*M*: maximum number of steps in the color map.

*m*: minimum number of steps in the color map.

Here, we made 64 steps color map. So, M = 64, m = 0.

The algorithm we used was implemented in MATLAB R2018a.

## Results

### Similarities in B-scan images

**Figure 2:**
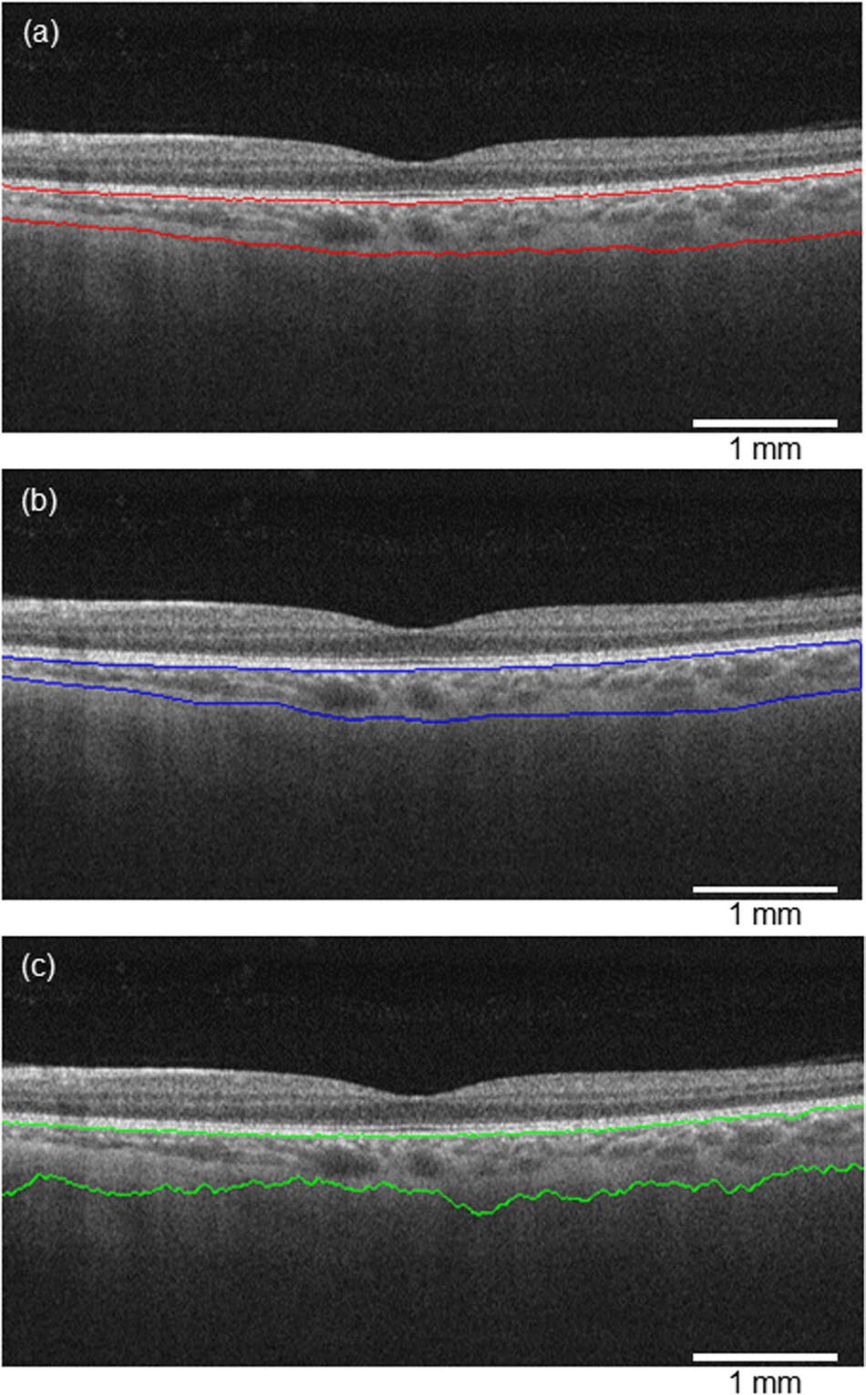
Segmentation results. The semantic segmentation (**a**), manual segmentation (**b**), and graph cut segmentation (**c**). The choroidal area segmented by the semantic segmentation contains a portion thought to be the posterior stromal layer that is seen in the manual segmentation.

We compared the results of the semantic segmentation (SegNet) to manual segmentaion and that made by graph cut method (Fig. 2). The SegNet model showed a similar segmentation to the manual segmentation, whereas the graph cut method partially failed to delineate the C-S boundary. Table 1 shows the areas measured using each method. We analyzed the DSC between the segmentation by three manual graders (A, B, and C) individually, the SegNet model, and the graph cut method (Table 2). The median of intergrader similarity between the three manual segmentations was 0.9184–0.9358. All of the DSC medians between the SegNet and manual segmentation by each grader were above 0.9000, meaning that the automated segmentation of the choroid using SegNet was not inferior to manual segmentation. The DSC median between the graph cut method and manual segmentation was low in each manual segmentation compared to that using SegNet.

**Table 1:** The area segmented in 290 B-scans.

(mm2)	Mean	S.D.	Median	Range
SegNet	1.9368	0.3110	1.9337	1.2983–2.7622
Graph cut	2.4586	0.3205	2.4499	1.6229–3.6707
Grader A	1.8416	0.3195	1.8706	1.1820–2.8368
Grader B	1.8513	0.4053	1.8613	1.0761–2.9485
Grader C	1.7604	0.3431	1.7373	1.0413–2.6863

**Table 2:** The median similarity indices (DSC) (upper) and similarity index ranges (lower) among segmentation methods.

	Graph cut	Grader A	Grader B	Grader C
SegNet	0.8306 0.6128–0.9507	0.9494 0.7232–0.9795	0.9447 0.8214–0.9723	0.9275 0.8430–0.9730
Graph cut		0.8101 0.5408–0.9527	0.8059 0.5614–0.9373	0.7877 0.5116–0.9342
Grader A			0.9282 0.6829–0.9762	0.9358 0.7738–0.9752
Grader B				0.9184 0.8163–0.9732

### Reproducibility

**Figure 3:**
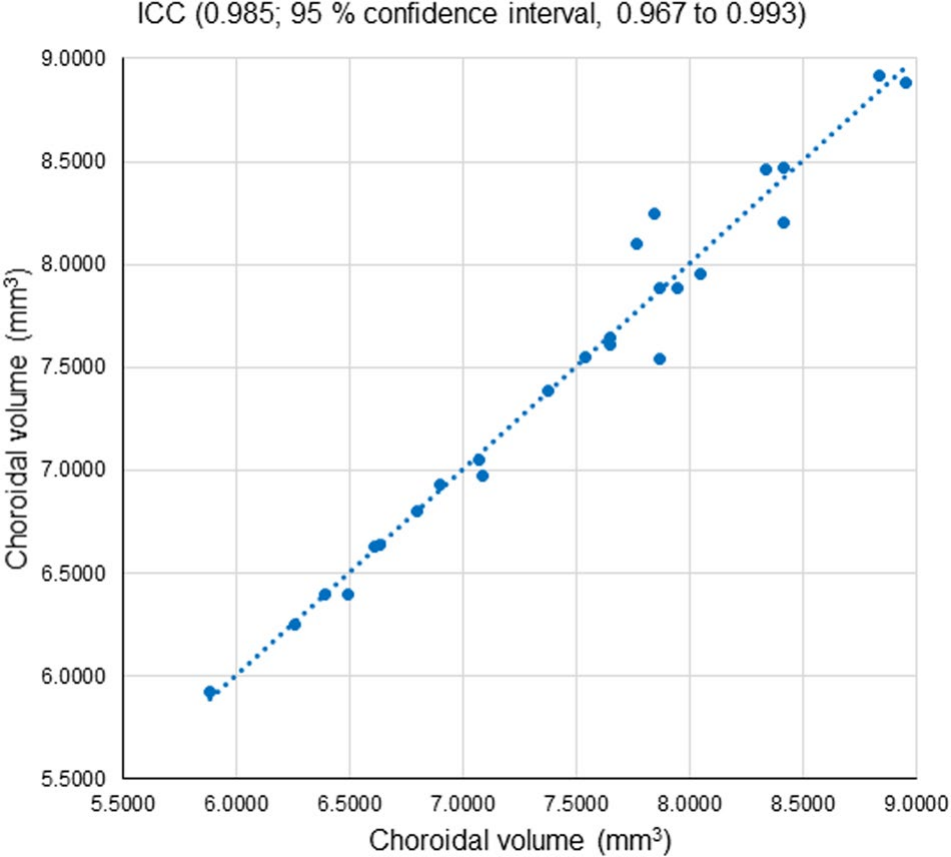
The intraclass correlation coefficient (ICC) between the repeatedly measured two choroidal volumes in twenty-five eyes. The mean volume was 7.4671 mm^3^ (standard deviation, 0.8168).

We were able to measure fifty choroidal volumes of all twenty-five pairs of cube scans using SegNet. The ICC between the choroidal volumes in the repeatedly measured two images was 0.985 (95% confidence interval, 0.967 to 0.993) (Fig. 3). The mean choroidal volume in the posterior pole (5.4 × 4.8 mm) was 7.4671 ± 0.8168 (standard deviation) mm^3^ in twenty-five healthy eyes. We analyzed the DSC of the twenty-five pairs of choroidal volumes calculated using SegNet. The DSC median was 0.9090 (standard deviation, 0.0554; range 0.7650–0.9647).

### 2D thickness map

**Figure 4:**
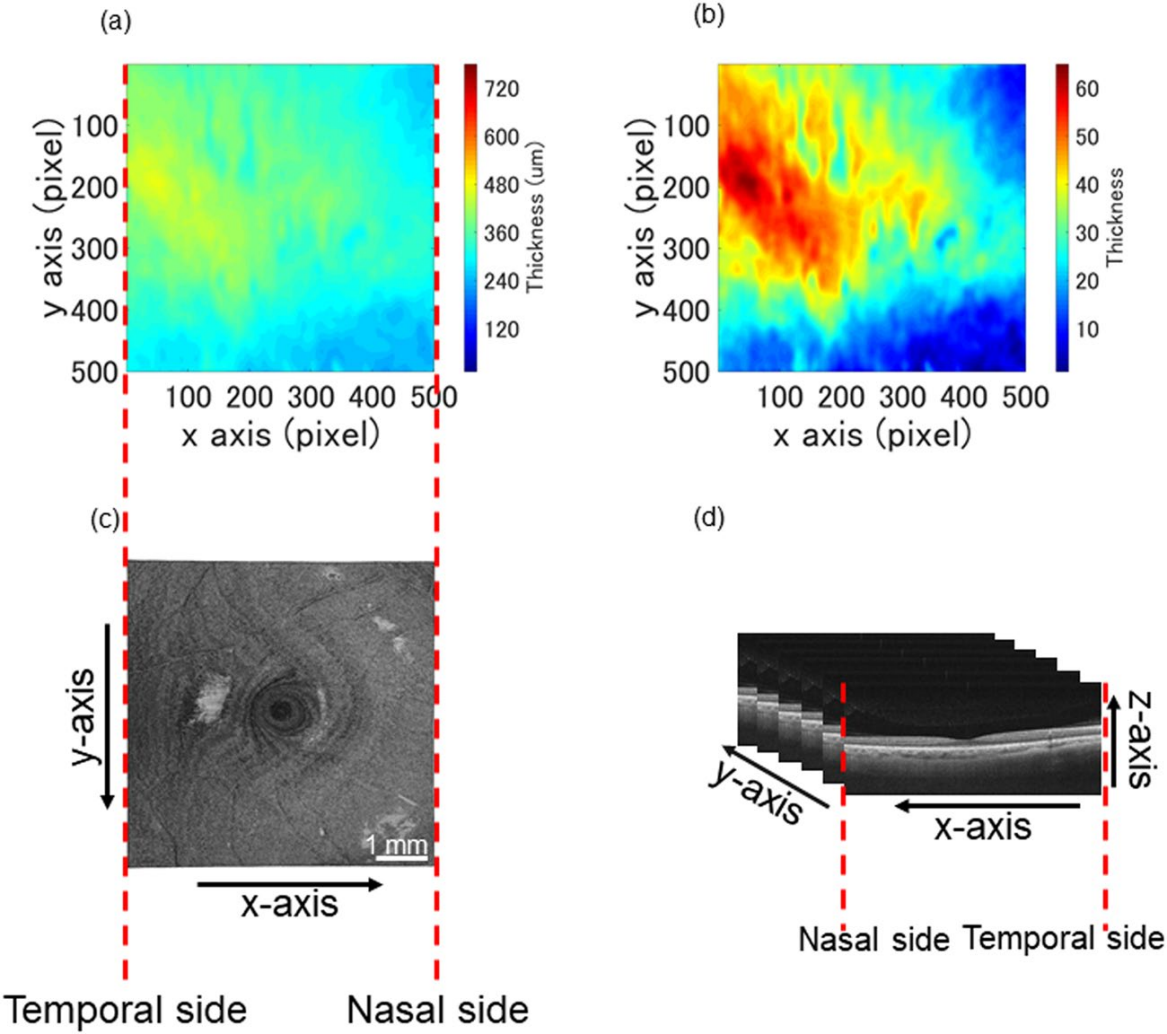
2D choroidal thickness maps of a 32-year-old man. The original 2D choroidal thickness map (**a**) and that after normalization (**b**). The map indicates that the thickest part of the choroid was located temporal to the fovea center. An face fundus image of SS-OCT (**c**). The directions of the axes shows in (**d**). The left side in (**a**–**c**) indicates the temporal side.

Figure 4 shows 2D choroidal thickness maps before (a) and after normalization (b). The regional changes are enhanced in the normalized thickness map.

## Discussion

The median DSC among the SegNet and manual segmentation by each grader were 0.9494 (grader A), 0.9447 (grader B), and 0.9275 (grader C). The DSC medians among the manual segmentations were 0.9184 to 0.9358, and the SegNet results were almost equivalent to those of the manual segmentation (Table 2). The high ICC value (0.985) in the choroidal volumetrics suggested good reproducibility of SS-OCT measurement and 3D segmentation.

SS-OCT, which has a high penetration laser light source and provides depth-enhanced images, is suitable for imaging of the choroid. 3D image analysis and choroidal 2D maps using SS-OCT images are potentially useful to explore the unknown pathogenesis of the choroid. To accomplish this purpose, the choroidal segmentation has several problems that cannot be easily overcome; low contrast of the C-S boundary, a deficit of the inner surface of the sclera, namely scleral canals for vessels or nerves, and the presence of a posterior stromal layer and lamina fusca^27^.

Enhancement of the C-S boundary in the preprocessing of OCT images has been reported to improve the success rate of boundary detection^17,18,28^. Even if the C-S boundary is completely delineated, the sclera has a pathway through which blood vessels and nerves pass^28^. The pathway in the sclera should be corrected to make the outer surface of the choroid. Interpolation in the processing of the outer surface of the choroid^17^ can correct the deletion of the boundary. Since statistical image segmentation (maximally stable extremal regions) can be robust against scleral defects caused by the scleral canal, shadowing, and other elements of the choroid in the OCT images, software using these techniques has made it possible to successfully delineate the C-S boundary. However, in previous studies, the C-S boundary was delineated along the outer border of the choroidal vessels, detected using automated segmentation^17,29–31^. Recently, the choroidal posterior stromal layer has led to misinterpretations of C-S boundaries that have been delineated automatically^32^. In the current study, we trained the SegNet model to identify the outer edge of the posterior stromal layer, not the C-S boundary at the posterior limit of the choroidal vessel. Therefore, the SegNet achieved choroidal segmentation with high similarity to manual segmentation under the same rule, which was superior to the graph cut segmentation.

An additional advantage of the SegNet model is that it was able to successfully delineate the boundary (Fig. 2) without denoising or edge enhancement, although the previous segmentation model required edge enhancement or inclination adjustment^17,31^.

Limitations. The present study has several limitations. First, in this series, we did not examine eyes with retinal and choroidal diseases, such as diabetic maculopathy or AMD, so we do not know whether our method can segment the choroids of such eyes effectively. If not, additional training using images of eyes with diseases can improve the segmentation results in such eyes, since the DCNN model is versatile. Second, the parameters to make 3D spline can influence the volume of the choroid, especially in eyes with an irregular C-S boundary, such as in CSC^10,33^ or polypoidal choroidal vasculopathy^34^. Third, histopathological correlation to SS-OCT images was not validated; we were unable to identify whether C-S boundaries segmented by SegNet or human grader in SS-OCT images matched histopathologically segmented borders. The definition of the C-S boundary on the SS-OCT should be made based on histopathological studies in the future.

### Conclusions

Semantic segmentation of SS-OCT images using DCNN is comparable to manual segmentation with high reproducibility.
